# Bacteriophage T4 as a Protein-Based, Adjuvant- and Needle-Free, Mucosal Pandemic Vaccine Design Platform

**DOI:** 10.1146/annurev-virology-111821-111145

**Published:** 2024-08-30

**Authors:** Jingen Zhu, Pan Tao, Ashok K. Chopra, Venigalla B. Rao

**Affiliations:** 1 Bacteriophage Medical Research Center, Department of Biology, The Catholic University of America, Washington, DC, USA; 2 State Key Laboratory of Agricultural Microbiology, College of Veterinary Medicine, Huazhong Agricultural University, Wuhan, Hubei, China; 3 Department of Microbiology and Immunology, Sealy Institute for Vaccine Sciences, Institute for Human Infections and Immunity, and Galveston National Laboratory, University of Texas Medical Branch, Galveston, Texas, USA

**Keywords:** needle- and adjuvant-free intranasal vaccines, bacteriophage T4 assembly, CRISPR engineering, pandemic vaccine design, broad humoral immunity, cellular immunity, mucosal immunity

## Abstract

The COVID-19 pandemic has transformed vaccinology. Rapid deployment of mRNA vaccines has saved countless lives. However, these platforms have inherent limitations including lack of durability of immune responses and mucosal immunity, high cost, and thermal instability. These and uncertainties about the nature of future pandemics underscore the need for exploring next-generation vaccine platforms. Here, we present a novel protein-based, bacteriophage T4 platform for rapid design of efficacious vaccines against bacterial and viral pathogens. Full-length antigens can be displayed at high density on a 120 × 86 nm phage capsid through nonessential capsid binding proteins Soc and Hoc. Such nanoparticles, without any adjuvant, induce robust humoral, cellular, and mucosal responses when administered intranasally and confer sterilizing immunity. Combined with structural stability and ease of manufacture, T4 phage provides an excellent needle-free, mucosal pandemic vaccine platform and allows equitable vaccine access to low- and middle-income communities across the globe.

## 1. INTRODUCTION

Historically, vaccine design is a long and arduous process that takes decades from discovery to manufacture, regulatory approval, and global distribution. Three types of vaccines are most commonly used: whole pathogen vaccines, subunit vaccines, and virus-like particle (VLP) vaccines (1). Whole pathogen vaccines, such as whole-cell pertussis and *Bacillus* Calmette-Guérin (BCG) vaccines (2, 3), can pose risks, especially to those individuals with compromised immune systems, due to the inclusion of live-attenuated or inactivated pathogens. The subunit vaccines contain only the well-defined target antigens produced in a recombinant expression system, but they may not be highly immunogenic, thus requiring formulation with chemical additives often referred to as adjuvants (e.g., Matrix-M for Novavax’s NVX-CoV2373 COVID-19 vaccine) (4). The VLP vaccines are nanometer-scale particles, either viral capsid shells or synthetic material, which display multiple antigen molecules on the surface [e.g., human papillomavirus (HPV) vaccine] (5). Most VLPs are restricted to a particular disease and still require adjuvants for stimulating strong immune responses.

When the SARS-CoV-2 virus emerged in December 2019, these traditional vaccine designs proved inadequate. The virus, causing COVID-19, quickly became a global pandemic by March 2020. Newer vaccine design strategies to rapidly produce vaccines on a massive scale were desperately needed to blunt the morbidity and mortality. This led to the development of lipid nanoparticle (LNP) encapsulated mRNA vaccines and adenovirus (Ad)-vectored DNA vaccines at an unprecedented speed (6, 7). By November 2020, just 9 months after the pandemic’s declaration, Pfizer’s and Moderna’s phase 2/3 trials showed that their mRNA vaccines provided ~95% protection against severe COVID-19 disease (8).

In parallel, various other vaccine designs emerged, but only LNP-mRNA, Ad-vectored, and inactivated viral vaccines could meet the global demand for billions of doses (9). While this extraordinary achievement transformed vaccinology research, it also exposed certain limitations of these platforms. These include insufficient breadth and durability of immune responses, lack of mucosal immunity, and challenges of breakthrough infections due to emerging viral variants (10–13). Additionally, these vaccines, particularly the mRNA vaccines, are temperature sensitive and costly, with about 65% of people in low-income areas yet to be vaccinated (14). More efficacious, inexpensive, and thermostable vaccine platforms are needed to fill these gaps and to effectively mitigate future pandemic threats at a global scale.

Here, we have presented one such potential pandemic vaccine platform using bacteriophage (phage) T4, a virus that infects *Escherichia coli* bacterium. Phage T4 is one of the most efficient viruses and has served as an extraordinary model in molecular biology. Structures of most of the components of T4 phage have been determined to near-atomic resolution; its genetics has been extensively investigated; and the mechanisms of assembly, genome packaging, and infection are well understood (15–17). Certain features of phage T4 allow the incorporation of hundreds of antigen molecules into its 120 × 86–nm capsid structure. These, in the past ~30 years of translational research (18, 19), led to the development of phage T4 as a versatile platform for vaccine design against diverse bacterial and viral pathogens (20). Efficacious vaccines have been developed against two of the deadliest biothreat diseases, inhalation anthrax caused by *Bacillus anthracis* and pneumonic plague caused by *Yersinia pestis*, as well as against pandemic COVID-19 and Flu. Remarkably, the T4-based vaccines are efficacious not only through traditional intramuscular administration but also through the intranasal route, eliciting potent and broad immune responses including mucosal immunity in mouse models (20–25). With a highly stable structure and relative ease of manufacture, phage T4 can serve as a universal next-generation platform for rapid production of vaccines on demand, either monovalent or multivalent, in response to an epidemic or pandemic, which can potentially be distributed globally without a cold-chain requirement (21, 23).

In this review, we present a detailed evaluation of the phage T4 vaccine technology with a particular focus on its unique architecture, engineering models, immunological responses, and potential as a protein-based, mucosal vaccine platform. We suggest that phage T4 provides an excellent complementary scaffold for vaccine development to better prepare the world against future deadly pandemic and bioweapon threats. Also, the goal is to provide equitable vaccine access to low- and middle-income communities across the globe.

## 2. VACCINE-INDUCED IMMUNE RESPONSES

**Figure 1:**
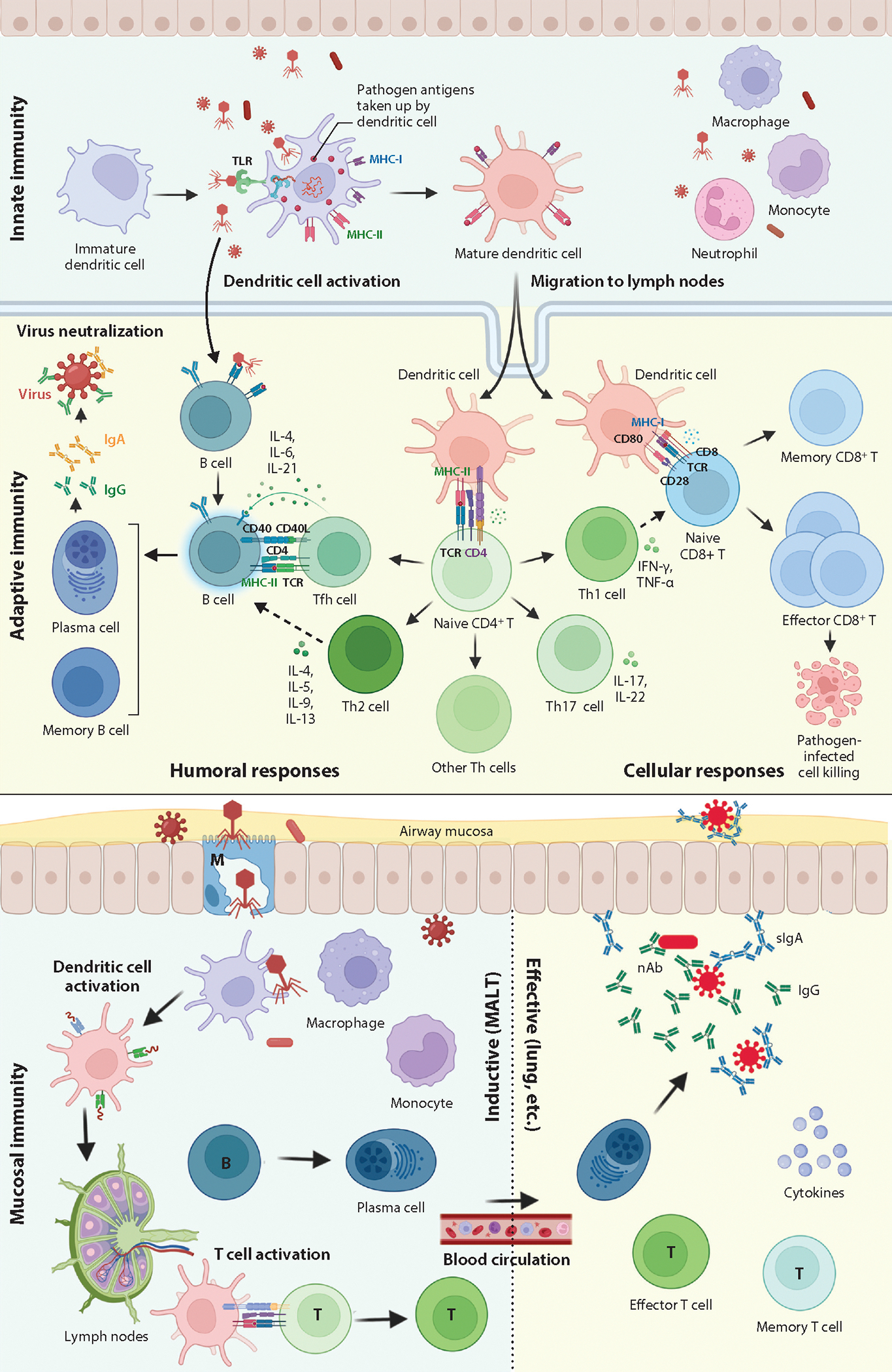
Vaccine- or pathogen-induced immunological pathways: innate, adaptive, and mucosal immune responses. The top section shows the innate immunity, including the transition of an immature dendritic cell upon vaccine or pathogen detection to a mature form and its migration to lymph nodes. The middle section shows the adaptive humoral and cellular responses. B cells, T cells, and various subtypes and interactions are depicted, alongside the cytokines released. The bottom section depicts mucosal immunity involving dendritic cells, B cells, T cells, and production of secretory IgA antibodies at the airway mucosa. Abbreviations: MALT, mucosa-associated lymphoid tissue; MHC, major histocompatibility complex; nAb, neutralizing antibody; TCR, T cell receptor; TLR, Toll-like receptor. Figure adapted from images created with BioRender.com.

An ideal vaccine should elicit multifaceted immune responses and afford complete protection against pathogen invasion, preferably providing sterilizing immunity. The responses include innate immunity for immediate defense, adaptive immune responses with memory for short- and long-term protection, and mucosal immunity to block the transmission of pathogens at the portal of entry (1) (Figure 1).

### 2.1. Innate Immune Responses

The innate immune system initially defends against pathogens (or vaccines) by identifying conserved molecular patterns, known as pathogen-associated molecular patterns (PAMPs) (26), using pattern recognition receptors (PRRs) such as the Toll-like receptors (TLRs) found in various immune cells such as dendritic cells (DCs) and macrophages (27). These trigger cell activation, releasing inflammatory cytokines (28) and creating an inflammatory microenvironment that attracts additional immune cells to the infection site (29).

DCs are key in this process as professional antigen-presenting cells (APCs), linking innate and adaptive immunity (30). They internalize antigens through mechanisms such as endocytosis, phagocytosis, and pinocytosis, and then they process and present these antigens to T cells (31). Activated DCs upregulate their antigen processing capabilities and increase the expression of major histocompatibility complex (MHC) molecules for antigen presentation (30). Mature DCs then migrate to lymph nodes to activate T cells specific to the antigens (31) (Figure 1).

### 2.2. Adaptive Immune Responses

Adaptive immune responses are initiated in lymph nodes when mature DCs present antigen epitopes to naive T cells. Naive CD8^+^ T cells recognize antigens on MHC class I, differentiating into memory cells and cytotoxic T lymphocytes that eliminate infected cells (32). Naive CD4^+^ T cells recognize antigens on MHC class II, differentiating into memory cells and T helper cells including Th1, Th2, Th17, Tfh, etc., depending on the cytokine milieu (32, 33). Th1 cells aid in cell-mediated immunity against intracellular pathogens; Th2 in promoting humoral antibody-mediated immunity against extracellular pathogens (34, 35); Th17 in generating inflammation and defenses against extracellular bacteria and fungi, especially at mucosal barriers (36); and Tfh in assisting B cells for antibody production and memory B cell formation (32) (Figure 1). B cells bound to vaccine antigens then proliferate with or without CD4^+^ Tfh cell assistance and form germinal centers. Here, they undergo class switching and somatic hypermutation for higher-affinity antibodies and differentiate into either plasma cells or memory B cells (37). Plasma cells secrete high-affinity antibodies to neutralize or mark pathogens for destruction (38). Memory B cells stay in circulation, lymph nodes, and bone marrow, enabling rapid antibody response recall (39) (Figure 1).

### 2.3. Mucosal Immune Responses

Mucosal surfaces such as the respiratory, gastrointestinal, and urogenital tracts, which are the entry points for pathogens such as SARS-CoV-2 and influenza, are protected by mucosal immunity (40, 41). This first line of defense is linked to the systemic immune system via circulating antibodies and lymphocytes. The mucosa-associated lymphoid tissue (MALT) in these tracts is rich in immune cells and lymphoid follicles and the key for eliciting innate and adaptive mucosal immunity (42).

The mucosal epithelium acts as a physical barrier and facilitates antigen sampling by APCs and M cells.M cells transport antigens to immune cells in the MALT, triggering B and T cell activation (42). Activated B cells then move to the lamina propria where they differentiate into IgA-secreting plasma cells. IgA binds to receptors on epithelial cells and is transported to mucosal surfaces as secretory IgA (sIgA) (43). This sIgA coats pathogens, preventing infection of mucosal epithelial cells as well as reducing viral/bacterial shedding after infection, thereby blocking pathogen transmission (44) (Figure 1).

MALT also contains large populations of T cells, including CD4^+^ T helper cells aiding IgA class switching and CD8^+^ cytotoxic T cells with effector functions (42). Additionally, tissue-resident memory T cells (T_RM_) are enriched at mucosal sites including the lungs and the intestine. CD8^+^ T_RM_ cells mediate rapid local antiviral immunity on pathogen re-exposure by releasing cytokines and chemokines, while CD4^+^ T_RM_ cells help maintain CD8^+^ T_RM_ and B cells (32) (Figure 1).

Collectively, the mucosal immune system, centered on MALT, protects mucosal surfaces through elicitation of multi-pronged antibody- and cell-mediated responses. Leveraging this in vaccine design can block pathogen entry and exit, thus minimizing infection and transmission by inducing mucosal sterilizing immunity. Vaccines triggering mucosal responses, especially via intranasal (i.n.) vaccination, are therefore highly effective against respiratory pathogens when compared to traditional intramuscular (i.m.) vaccines that do not induce significant mucosal immunity (11).

## 3. BACTERIOPHAGE T4 AS A UNIVERSAL NANOVACCINE PLATFORM

### 3.1. Immunostimulatory Mechanisms of Phages

Phages show promise as versatile vaccine platforms due to their nanoscale size, particulate nature, overall safety, and low cost of manufacture (45). They have generally been shown to stimulate robust immune responses through several possible mechanisms. First, the nanoscale size of phage particles, typically 20–200 nm, facilitates transport within the mammalian body. Smaller phages efficiently drain through lymphatics into lymph nodes for immune sampling. Larger phages require APC-mediated transport (29). Additionally, their particulate nature promotes phagocytosis and cross-presentation to stimulate CD4^+^ and CD8^+^ T cells (46).

Second, phage capsids, referred to as VLPs, share structural similarities with eukaryotic viruses. The repetitive and symmetric arrangement of capsid structural proteins resembles PAMPs of human viruses that activate PRRs. For example, the HK97 fold of phage capsid proteins and the dodecameric structure of portal vertices are well conserved in *Caudoviricetes* phages and *Herpesviricetes* viruses (47, 48). These features likely engage and cross-link extracellular PRRs such as TLR2 (49, 50), triggering inflammatory signaling. Intracellularly, phagocytosed and processed capsids may stimulate responses through binding to endosomal TLRs (51).

Third, phages contain genomic DNA and RNA that can be detected by several PRRs such as TLRs 3, 7, 8, and 9 (52, 53). TLR3 recognizes double-stranded (ds) RNA (54), TLR7 and TLR8 sense single-stranded RNA, and TLR9 detects unmethylated CpG motifs abundant in phage genomes (55). Additionally, the lack of 5′ mRNA capping and polyadenylation also marks phage RNA as nonself, which can be detected by cytosolic PRRs such as retinoic acid–inducible gene I (RIG-I) and melanoma differentiation-associated gene 5 (MDA5). The cytosolic nucleic acid sensors such as cyclic GMP-AMP synthase (cGAS) and interferon gamma–inducible protein 16 (IFI16) can detect phage DNA and activate stimulator of interferon genes (STING) and tank-binding kinase 1 (TBK1) (28). Activation of these receptors frequently leads to the production of proinflammatory cytokines (53, 56).

Fourth, the high copy number of major capsid proteins enables the modular incorporation of antigens into the phage particle (20). For example, the T4 phage capsid contains a combined total of more than 2,000 copies of exposed proteins that can display pathogen epitopes on the surface. Other phages such as λ and T7 harbor hundreds of capsid proteins that can display foreign epitopes (45). This multivalent display potently cross-links B cell receptors, triggering robust humoral immunity (57). Moreover, the copy number and spacing of displayed antigens can be tuned to optimize immunogenicity.

Fifth, phages might carry small amounts of bacterial lipopolysaccharide (LPS) on their surface because of the lysis of host bacteria in vivo, especially when administered via oral and intranasal mucosal routes (53). LPS strongly activates TLR4, leading to cytokine production and enhancing the downstream adaptive response (58).

Finally, phages can be internalized by human cells through various endocytic mechanisms, such as phagocytosis by immune cells, facilitated by receptors such as Fc, complement, and scavenger receptors (51, 59). Proteolytic processing and antigen presentation activate humoral and cell-mediated immunity. Additionally, phages can cross barrier tissues such as the gut and the respiratory airways, entering systemic circulation and reaching lymph nodes and spleen, further activating the immune system (60–62).

### 3.2. Phage T4 Capsid Architecture

Several features of the T4 capsid structure make it a highly flexible, plug-and-play vaccine design platform, particularly for intranasal vaccine delivery.

**Figure 2:**
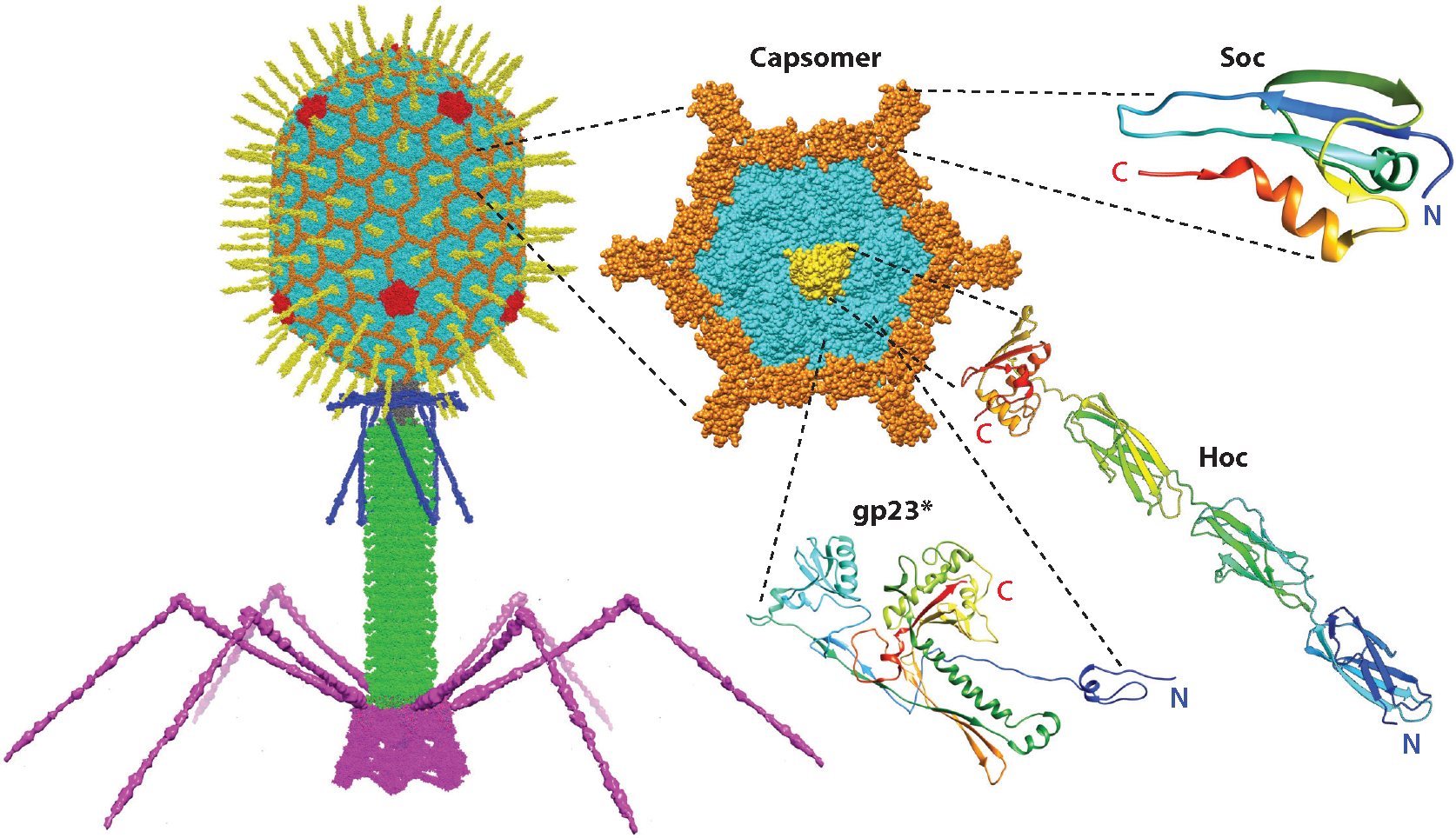
Structural components of bacteriophage T4 nanoparticle. One of the capsomers is enlarged to depict the structures and arrangement of major capsid protein gp23∗ (*cyan*), Soc (*brown*), and Hoc (*yellow*) subunits (N and C termini are labeled). Soc and Hoc are used for displaying pathogen antigens on the capsid surface. Abbreviations: Hoc, highly antigenic outer capsid protein; Soc, small outer capsid protein.

Phage T4 has three components: head (capsid), tail, and tail fibers. The 120 × 86–nm size prolate icosahedral capsid offers a large surface area for vaccine design and delivery (20, 63). It is assembled with 930 subunits (155 hexameric capsomers) of the major capsid protein gp23* (the asterisk represents the cleaved mature form) (49 kDa) with a defined surface lattice structure (64, 65) (Figure 2). Inside the head is an ~171-kb linear double-stranded DNA genome packaged through a dodecameric portal vertex (gp20, 61 kDa) by a powerful packaging motor (gp17, 69 kDa) (17, 66–68). The other 11 icosahedral vertices are occupied by the pentameric gp24* capsid protein (64, 65). The neck, tail, and fibers attach to the portal vertex in that order after completion of genome packaging to generate an infectious virion that exhibits one of the highest reported infection efficiencies (close to 100%) (69, 70).

A unique feature of the T4 capsid is that its large outer surface is decorated with two nonessential outer capsid proteins: 870 copies of the small outer capsid protein (Soc, 9 kDa) and 155 copies of the highly antigenic outer capsid protein (Hoc, 39 kDa). Soc assembles as trimers at quasi-threefold axes, clamping adjacent gp23* capsomers to impart additional stability to an already very stable capsid structure (71) (Figure 2). Consequently, T4 can withstand high temperature and pH exposures. Both the N and C termini of Soc are solvent exposed, making them suitable for gene engineering (72).

Hoc is a fiber consisting of a string of Ig-like domains. It binds at the center of each capsomer through its C-terminal domain, while the N-terminal domain is projected at ~180 Å away from the capsid (73, 74) (Figure 2).Although Soc and Hoc provide survival advantages for the T4 virus in natural environments (e.g., human gut, sewers), they are dispensable (75). Importantly, Soc and Hoc proteins bind to a capsid with exquisite specificity and nanomolar affinity, properties that are not compromised by the attachment of a foreign antigen to the termini of Soc and Hoc (76, 77). Indeed, the N and C termini of Soc and the N terminus of Hoc have been extensively utilized to generate high-density, symmetric arrays of foreign antigens displayed on the capsid surface (22, 76, 78, 79), including large proteins such as the anthrax protective antigen (PA) (~83 kDa) and the trimeric SARS-CoV-2 spike ectodomain (~435 kDa) (80).

Furthermore, Hoc possesses a natural ability to bind to mucin glycoproteins present in the nasal mucosal layer (81). This allows for efficient capture of T4 nanoparticles by the nasal mucosa, better uptake by mucosal-resident APCs, and greater persistence in the respiratory airways to stimulate robust and durable immune responses.

Additionally, up to ~1,000 molecules of the nonessential internal proteins (IPs I, II, and III) that are packaged inside the capsid along with the genomic DNA can also be replaced by foreign antigens (24, 82).

### 3.3. T4-Based Vaccine Design Strategies

**Figure 3:**
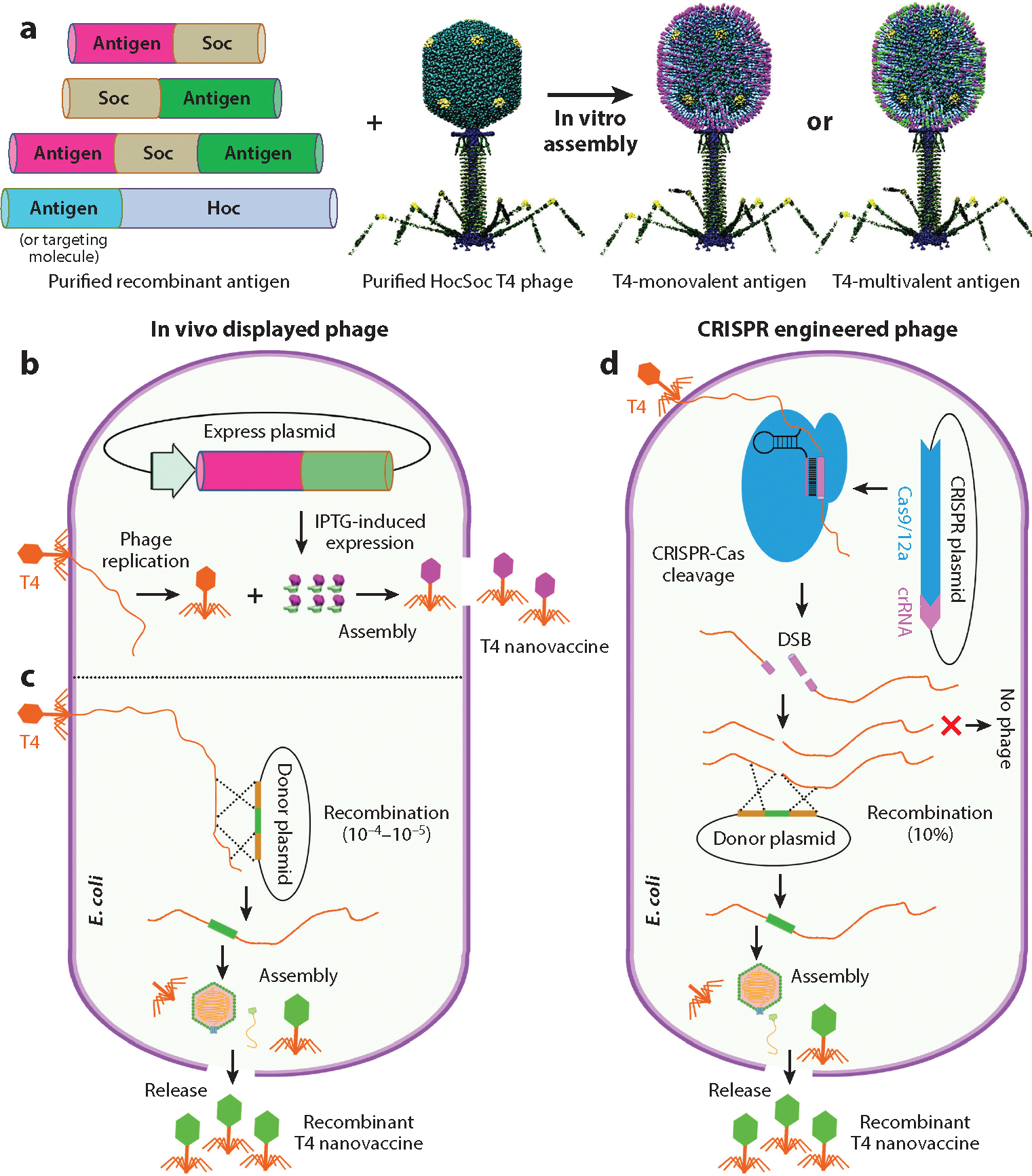
Phage display and engineering strategies. (*a*) In vitro assembly. Full-length antigens are fused at the N and/or C termini of Soc or Hoc, the fusion proteins expressed, purified, and displayed on capsid surface by simple mixing to generate monovalent and multivalent vaccines. (*b*) In vivo assembly. Soc- or Hoc-fused antigens are shown on the T4 capsid following their expression in *Escherichia coli* from a recombinant plasmid. The expression is driven by an IPTG-inducible phage T7 promoter. (*c*) Recombination and in vivo assembly. The Soc- or Hoc-fused antigen gene is incorporated into the T4 genome via homologous recombination. The recombinant phage expresses the fusion protein during infection, which is assembled on the capsid. (*d*) CRISPR-engineered phage. Same as in panel *c* except that the CRISPR-Cas machinery is used to introduce targeted double-strand breaks into the T4 genome for efficient recombination with a donor plasmid. Abbreviations: crRNA, CRISPR RNA; DSB, double-strand break; Hoc, highly antigenic outer capsid protein; IPTG, isopropyl-β-d-thiogalactopyranoside; Soc, small outer capsid protein.

The above architectural features and the well-studied mechanisms of assembly provide exceptional opportunities for developing T4 as a universal vaccine design platform. We and others developed several approaches to incorporate pathogen antigens into the T4 nanoparticle (Figure 3).

#### 3.3.1. In vitro assembly.

Many studies have shown that antigens and oligomeric complexes belonging to various infectious diseases including COVID-19, Flu, anthrax, plague, and AIDS can be efficiently displayed on the T4 capsid by in vitro assembly (21–25, 80, 83, 84). This is achieved by tethering antigens to capsid through fusion to Soc and/or Hoc proteins with an affinity tag, up to 1,025 molecules per capsid. The proteins are expressed in *E. coli* or mammalian cells, purified by affinity chromatography, and loaded onto T4 capsid by simple mixing of antigen(s) and *hoc*^−^ *soc*^−^ phage at an appropriate ratio (79) (Figure 3a). By incorporating SpyTag/SpyCatcher (85), the antigens could also be covalently tethered to capsid as has been demonstrated in the case of SARS-CoV-2 spike trimers (24).

The in vitro display strategy has several advantages. First, it allows for the display of functionally characterized, native-like antigens that can trigger potent protective immune responses. Second, it allows efficient display of large full-length proteins [e.g., 56-kDa *Y. pestis* F1mutV (monomeric form of capsular antigen F1 and low calcium response V antigen) (79), 83-kDa PA (76) of *B. anthracis*], oligomeric complexes [433-kDa SARS-CoV-2 spike trimer (24), 600-kDa tripartite anthrax toxin complex (86)], and even a whole virus [3.8-MDa, ~25-nm adeno-associated virus (AAV) particle (80)]. Additionally, the composition of in vitro assembly can be adjusted to control antigen copy number and to develop multivalent vaccines (76).

#### 3.3.2. In vivo assembly.

In vivo display involves pre-expression in *E. coli* of Hoc-/Soc-fused antigens from a recombinant plasmid or bacterial genome using a strong promoter such as the T7 promoter followed by infection with *hoc*^−^ *soc*^−^ mutant phage and display (24, 87). This approach provides better control over expression but may be subject to potential proteolytic cleavage and/or aggregation of the antigen (24, 87) (Figure 3b).

Another in vivo assembly approach is to incorporate the antigen into the capsid interior. Each T4 capsid contains ~1,000 molecules of three nonessential IPs I, II, and III packaged along with the genome, all of which can be potentially replaced with pathogen antigens (88). Guided by a 10-residue capsid targeting sequence (CTS) at the N terminus, the IPs are assembled as part of the scaffolding core around which the capsid subunits assemble to form the icosahedral shell. Although the scaffolding core is degraded by a maturation protease (gp21) to create space for genome packaging, the highly basic IPs or any foreign proteins fused to the CTS in place of IPs remain in the capsid (82, 88). A variety of proteins including the SARS-CoV-2 nucleocapsid protein (NP) (24, 88, 89) have been encapsulated using this approach.

#### 3.3.3. CRISPR engineering.

In an alternative in vivo display approach, the antigen gene is inserted into the T4 genome through fusion to *hoc* or *soc* gene by homologous recombination with a recombinant plasmid. The recombinant phage during infection of *E. coli* would express the fusion protein, which is then assembled on the phage capsid (19). However, the recombinant frequency, even for the highly recombinogenic T4, is low, ~10^−4^ to 10^−5^ relative to parental phage background, necessitating laborious screening and selection protocols (18) (Figure 3c).

We have developed a powerful CRISPR engineering strategy that allows efficient and targeted insertion of antigen gene(s) into the phage genome (90–92). This is particularly useful in epidemic and pandemic situations for rapid screening of multivalent vaccine candidates to select the most effective one for vaccine production (21, 23, 24). Accordingly, in response to the COVID-19 pandemic, we constructed dozens of vaccine candidates incorporating SARS-CoV-2 structural components including the ~6.5-kb full-length spike S gene, the 2.7-kb receptor binding domain (RBD) gene, and a 1.3-kb nucleocapsid gene, along with Hoc- and Soc-fused SARS-CoV-2 genes controlled by the Hoc and/or Soc native promoters in a matter of weeks (23, 24, 72).

The CRISPR strategy involves the construction of *E. coli* strains carrying two recombinant plasmids: the “spacer” plasmid, encoding a genome-editing nuclease (type II Cas9 or type V Cas12a) and CRISPR RNAs (crRNAs or spacer RNAs) (93–95), and the “donor” plasmid, containing the antigen gene flanked by ~500-bp homologous arms of phage genome. When these *E. coli* are infected by the T4 phage, the genomic DNA is cleaved at the protospacer sequence, resulting in loss of phage propagation. However, recombinants that integrate the antigen gene into the phage genome survive with a remarkable efficiency of ~10% (24, 90, 91) (Figure 3d), far surpassing the ~10^−4^–10^−5^ efficiency seen with traditional genetic approaches (18). The progeny phage then harbors the antigen genes, which are subsequently expressed in *E. coli* and/or human cells, depending on the controlling promoter.

Given the circularly permuted and terminally redundant nature of the T4 genome (96), any insertion exceeding ~3 kb may result in a loss of terminal redundancy and infectivity. To facilitate the incorporation of larger antigenic gene segments, a series of acceptor phages with deletions ranging from 7 to 30 kb of the nonessential genome have been constructed using CRISPR (24, 72). This strategic adaptation enabled the successful insertion of multiple pathogen gene segments from SARS-CoV-2 and influenza virus (see Sections 4.1 and 4.2) (21, 23, 24).

#### 3.3.4. Prime-boost vaccine design.

The T4 head alone—devoid of tail, neck, and genome—provides a unique platform for simultaneous delivery of DNA and protein antigens in a single immunization (63, 77, 78, 80, 97). This system enables custom incorporation of antigen genes into capsid through in vitro DNA packaging, which could then be decorated with Soc- and Hoc-fused antigens and cell-targeting molecules (77, 78, 80). The displayed antigen might prime the immune system while the antigen continuously expressed from DNA for a period of time might boost the immune responses. The 180-Å-long Hoc fibers are particularly suitable for targeted vaccine delivery to antigen-presenting DCs by attaching ligands such as DEC205 monoclonal antibody or CD40 to the N-terminal tip (77). This was demonstrated for plague F1-V antigen and Flu hemagglutinin (HA) stem domain, which elicited both humoral and cellular responses and notably durable antibody responses in mice that remained high even after 6 months (77, 80).

## 4. T4-ENGINEERED VACCINES

The T4 phage platform has been studied over the past 25–30 years, to design efficacious vaccines against deadly viral and bacterial diseases including COVID-19, Flu, anthrax, and plague (Table 1).

### 4.1. The T4-COVID Vaccine

**Figure 4:**
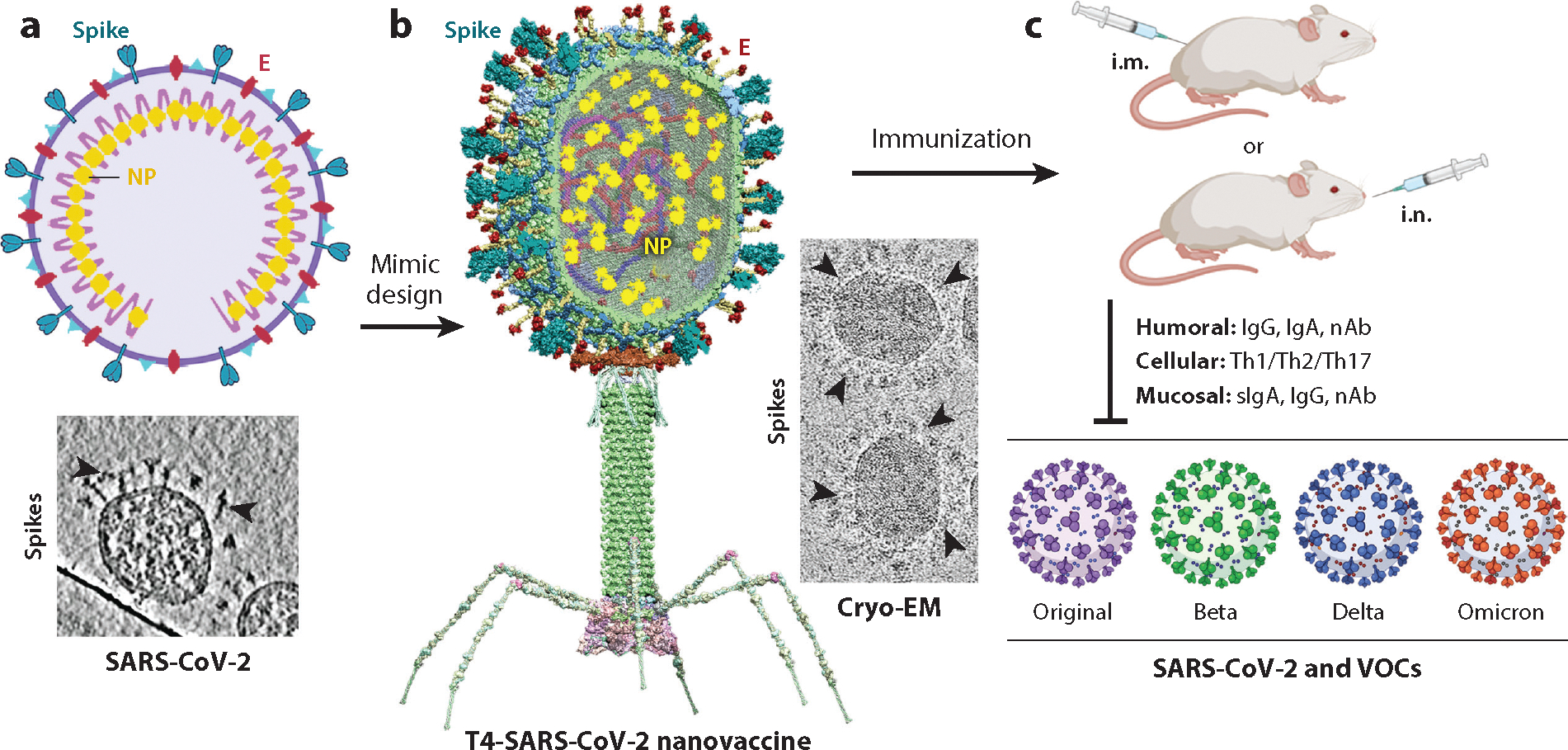
Intranasal immunization elicits broad immune responses including mucosal immunity. (*a*) Top: schematic of the SARS-CoV-2 virus, highlighting key structural components: spike, E, and NP. Bottom: cryo-EM image shows spike trimers on the virion surface (98), marked by arrowheads. (*b*) Design of the T4-SARS-CoV-2 nanovaccine displaying the vital structural components of the CoV-2 virus. The cryo-EM image shows displayed spike trimers, marked by arrowheads, mimicking the spike trimers on CoV-2 virion. (*c*) Immunization with the T4-COVID vaccine, either through i.m. or i.n. route, elicited robust humoral responses (IgG, IgA, nAb), cellular responses (Th1, Th2, Th17 responses), and in addition, through i.n. route, mucosal responses (sIgA, IgG, nAbs). The nAbs neutralize SARS-CoV-2 and its VOCs (Beta, Delta, and Omicron). Abbreviations: cryo-EM, cryo–electron microscopy; i.m., intramuscular; i.n., intranasal; nAb, neutralizing antibody; NP, nucleocapsid protein; VOC, variant of concern.

When reports of SARS-CoV-2 infections emerged in January 2020, we embarked on designing a multivalent COVID-19 vaccine by incorporating three key structural components of the virus: spike, envelope protein, and NP (24, 72). In about 8 months, our research progressed from concept to candidate, from construction and biochemical analysis of dozens of vaccine candidates to vaccine production and testing in mice (Figure 4).

The most efficacious T4-COVID vaccine contained the spike trimer and the external domain of envelope (Ee) protein displayed on capsid and the NP packaged in the capsid interior (24). The spike trimers were fused to a 16-aa SpyTag and covalently tethered to capsid through Soc-SpyCatcher whereas the Ee peptides were directly fused to Hoc. In subsequent studies, it was clear that spike trimer alone is sufficient for protection. Cryo–electron microscopy showed spike trimers displayed in a native-like configuration on the capsid surface, apparently presenting the epitopes in a native-like context (24, 98) (Figure 4a,b).

The resultant T4-COVID vaccine is phage nanoparticles suspended in phosphate-buffered saline (PBS) with no adjuvants or added chemicals. When administered intramuscularly or intranasally, the vaccine elicited broad antigen-specific immune responses in mouse and rabbit models. These include neutralizing antibody (nAb) responses, cellular responses, and mucosal sIgA responses (23, 24) (Figure 4c).

#### 4.1.1. Humoral immune responses.

The T4-COVID vaccine induced balanced CoV-2-specific Th1 (IgG2a) and Th2 (IgG1) subtype antibody responses (23, 24), in contrast to traditional subunit-alum vaccines that exhibit a Th2-bias. Stimulation of both Th1 and Th2 immunity is desirable because Th1 contributes significantly to long-lasting protection (99). Furthermore, the T4-COVID vaccine also triggered high levels of serum IgA antibodies via both i.m. and i.n. routes of administration (23), a response typically not seen with traditional vaccines (100). Given the anti-inflammatory activity of sIgA and its superior early-phase viral neutralization compared to IgG (101), the production of IgA antibodies might also be a desirable feature.

The T4-COVID vaccine-induced antibodies demonstrated broad neutralization of SARS-CoV-2 and its variants of concern (VOCs), including ancestral WA-1/2020, Beta (B.1.351), Delta (B.1.617.2), and Omicron (B.1.1.529) variants (23, 24). While Delta is notably contagious and more deadly, Beta exhibits robust immune evasion, and Omicron combines a high rate of transmission with significant vaccine evasion (9). The nAb titers were comparable among the WA-1/2020, Beta, and Delta, but six times lower against Omicron (23), mirroring a trend also observed with the mRNA vaccines (102). However, Omicron neutralization of bronchoalveolar lavage fluids (BALFs) was comparable to that of the WA-1/2020 strain (23). Moreover, remarkably, mice receiving i.n. immunization elicited 3 times more nAbs against SARS-CoV-2 and its VOCs than those immunized intramuscularly (23).

#### 4.1.2. Cellular immune responses.

The T4-COVID vaccine administered either i.n. or i.m. stimulated potent cellular immune responses in mice, including antigen-specific T cell proliferation and high percentages of CD4^+^ and CD8^+^ T cells positive for cytokines IFN-γ, TNF-α, and IL-17A (23). Again, remarkably, i.n. administration elicited significantly higher percentages of IFN-γ^+^ CD4^+^ and IFN-γ^+^ CD8^+^ T cells, as well as a substantial increase in Th1 cytokines (e.g., IFN-γ, IL-2, and TNF-α) when compared to i.m. administration (23). Because strong Th1 responses typically prevent vaccine-associated enhanced respiratory diseases, the T4-COVID vaccine that stimulated balanced Th1 and Th2 cellular immunity is also a desirable feature (103).

#### 4.1.3. Mucosal immunity.

The T4-COVID vaccine, when administered i.n., induced robust mucosal immunity (23), as evident by high titers of mucosal IgG and sIgA antibodies in BALF that are critical for protection from viral transmission at the portal of entry (11, 104). These responses, like the responses in serum, were also balanced between Th1 and Th2 subtype antibodies (23). On the other hand, i.m. administration failed to stimulate sIgA in BALF.

The T4-COVID vaccine also stimulated Th17 cellular immunity, which often collaborates with Th1 cells to protect against pathogen infections, especially in mucosal tissues (36), by boosting the production of antimicrobial peptides within the mucosal epithelium and recruiting neutrophils to eliminate pathogens (105). Thus, by eliciting Th1/Th2/Th17 and mucosal responses, the intranasal T4-COVID vaccination triggered multiple immune mechanisms for protection against COVID-19 (23).

#### 4.1.4. Broad protection and sterilizing immunity.

The T4-COVID vaccine provided complete protection against the mouse-adapted SARS-CoV-2 MA10 strain, the ancestral WA-1/2020 strain, and the most lethal Delta variant in both the conventional BALB/c and hACE2 knock-in mouse models (23, 24). It conferred sterilizing immunity as indicated by the lack of viral titer in the lungs of vaccinated and challenged mice. While control mice showed high lung viral loads (>10^7^ TCID_50_ per gm lung tissue on day 5), no live virus was detected in T4-COVID vaccinated mice post challenge (23). Furthermore, the T4-COVID vaccine showed minimal lung lesions, did not affect the gut microbiome, and remained stable at ambient temperature for at least the tested period of 10 weeks (23). This needle-free and adjuvant-free phage T4 mucosal vaccine might be able to minimize viral transmission by blocking viral acquisition and viral shedding, which requires further investigation.

### 4.2. The T4-Flu Vaccine

Flu, a respiratory illness caused by types A and B influenza viruses, is a major global health problem causing seasonal epidemics all over the world. Influenza viruses have segmented, single-stranded RNA genomes encoding 11–12 proteins (106). Type A has various subtypes determined by surface proteins, HA and neuraminidase (NA) (107). At least 18 HA and 11 NA subtypes exist, creating numerous combinations through reassortment during coinfections, necessitating annual vaccine updates to match the anticipated prevalent strains (108).

The primary target of vaccine-induced protective responses is the trimeric HA spike embedded in the viral envelope (108). HA is composed of two external domains: the relatively well-conserved stem domain that is not very immunogenic and a highly variable head domain that is immunodominant. Recombinant stem-only trimers without the head domain can stimulate broadly neutralizing antibodies that can confer heterosubtypic Flu virus immunity (109). Additionally, the conserved extracellular domain of the matrix protein 2 (M2e) elicits non-neutralizing yet broadly protective antibodies that mediate effector functions through antibody-dependent cell cytotoxicity (ADCC) (110).

We have incorporated both HA-Stem and M2e antigens into phage T4 to generate T4-Flu vaccine candidates (21, 80). Detailed studies were completed with the 3Me vaccine in which three M2e peptide variants from human, swine, and avian influenza viruses were fused in tandem with the Soc C terminus to induce broad immune responses against diverse Flu variants. The HA-stem-based vaccine candidates are under investigation.

#### 4.2.1. Systemic and mucosal immunity.

The Soc-3M2e protein was efficiently displayed on T4 capsids at high density either by in vitro assembly (25) or by in vivo assembly through CRISPR engineering (21). The resultant T4-(Soc-3M2e) vaccine is similar to the T4-COVID vaccine, with phage particles suspended in a simple PBS buffer with no adjuvants. Immunization of mice through i.m. and i.n. routes induced high titers of M2e-specific IgG antibodies in sera (21, 25). Unlike the soluble M2e, which primarily induced IgG1, T4-(Soc-3M2e) produced balanced levels of IgG1 and IgG2a subtypes, the latter being the key subtype for ADCC and phagocytosis that are essential for protection (110).Moreover, the T4-(Soc-3M2e) vaccine stimulated M2e-specific sIgA and IgG antibodies in BALF via the i.n. route, while the i.m. administration primarily stimulated the IgG antibodies (21, 25).

The T4-(Soc-3M2e) vaccine also stimulated strong M2e-specific cellular immune responses, demonstrated by increased IFN-γ and IL-4 secreting T cells in the spleen (25). Moreover, i.n. administration triggered robust M2e-specific CD4^+^ T cell responses in the lungs, including effector memory T (T_EM_) and T_RM_ subsets, which were not elicited upon i.m. administration (21). The generation of respiratory T_RM_ cells highlights the significance of the immunization route for inducing mucosal cellular responses that are essential for blocking the transmission of respiratory pathogens.

#### 4.2.2. Broad immunity and cross-protection.

The T4-(Soc-3M2e) vaccine, when administered intranasally, afforded complete protection against both M2e-homologous H1N1 (5LD_50_ of A/Puerto Rico/8/34) and M2e-heterologous H3N2 (3LD_50_ of A/duck/Shandong/12/2019) virus challenges in mice. However, i.m. vaccination protected only against the homologous H1N1 virus (21, 25). Vaccinated mice also showed no significant weight loss or lung pathology following the Flu virus challenge. These data demonstrate cross-protection through mucosal immunization.

Our studies showed that the T4-(Soc-M2e) i.n.-administered phage nanoparticles remained on airway epithelial surfaces for at least 26 days probably due to phage T4’s mucoadhesive properties conferred by the Hoc fibers (21). This sustained presence allows prolonged antigen exposure to various pulmonary APCs such as alveolar macrophages, DCs, and monocyte-derived DCs (31). These APCs likely uptake the T4-(Soc-M2e) particles through phagocytosis and endocytosis, facilitated by the particulate structure and immunostimulatory properties of the T4 phage (20). The antigen-loaded APCs undergo activation and maturation, eventually migrating to the lung-draining lymph nodes, aided by upregulated costimulatory molecules (21).

In the lymph nodes, APCs present M2e epitopes to naive CD4^+^ T cells, prompting their differentiation into effector and memory subsets. Some of these activated T cells return to the lungs, serving as T_RM_ cells, providing a frontline defense against Flu viruses (21). Moreover, in the lymph nodes, the interaction between Tfh cells and B cells leads to germinal center formation, class switching to IgG and IgA, and affinity maturation, all resulting in the secretion of high-affinity anti-M2e antibodies (21). The dimeric sIgA antibodies are transported to the respiratory lumen as the first line of mucosal humoral defense (21, 42).

Collectively, the data demonstrate that the T4-M2e nasal vaccine triggers potent mucosal and systemic immune responses, enabling viral clearance upon influenza exposure, preventing significant lung pathology or systemic spread, and protecting against diverse Flu variants. This vaccine is stable at 4°C for at least 10 months.

### 4.3. T4-Engineered Biodefense Vaccines

#### 4.3.1. The T4-anthrax vaccine.

Anthrax is a severe infectious disease caused by the spore-forming bacterium *B*. *anthracis*. It can infect humans and animals via gastrointestinal, cutaneous, or inhalation routes (111). Inhalation anthrax has a high mortality rate and is a major biothreat (111), as evidenced by the 2001 anthrax attacks. The Centers for Disease Control and Prevention (CDC) classifies *B. anthracis* as a Tier-1 biothreat agent given its extreme risk.

The major virulence factors of *B. anthracis* are the exotoxins composed of PA (83 kDa) and lethal factor (LF, 90 kDa) or edema factor (EF, 89 kDa) (112). The primary vaccine target is PA. The current Food and Drug Administration (FDA)-approved anthrax vaccines are AVA (anthrax vaccine adsorbed) and AVP (anthrax vaccine precipitated), which are acellular culture supernatants containing PA but not the other toxin components. Despite their efficacy, concerns over variability, safety, and dosing warrant the development of next-generation anthrax vaccines (113).

The T4 phage has been engineered as an anthrax vaccine through in vitro display (83). A series of vaccine candidates were constructed using Soc and/or Hoc to display PA alone or in complex with LF and EF. These have been successfully tested in New Zealand White rabbit and rhesus macaque models for protection against inhalation anthrax (84, 114). For instance, the T4-Soc-PA anthrax vaccine displaying ~355 PA molecules per phage was highly immunogenic in rabbits, inducing robust PA antibody titers and lethal toxin-neutralizing titers that are comparable to alum or liposome-adjuvanted recombinant PA vaccines (114). When challenged with *B. anthracis* Ames strain spores, 100% survival was achieved in both New Zealand White and Dutch belted rabbit models (22, 114). In the macaque model, T4-PA vaccination conferred complete protection when challenged with aerosolized lethal doses of *B. anthracis* Ames strain spores (84). Furthermore, the immunized macaques showed no bacteremia, lesions, tissue bacteria, or side effects (84).

#### 4.3.2. The T4-plague vaccine.

Plague is a highly infectious disease caused by the bacterium *Y. pestis*, also a CDC Tier-1 agent transmitted from rodents to humans via fleas (115). It causes bubonic, septicemic, and pneumonic plague disease with high mortality if untreated (116). *Y. pestis* caused three major pandemics including the Black Death, and it still causes thousands of cases annually (115). Although a vaccine has been a priority, there is no FDA-approved vaccine yet. The capsular antigen fraction 1 (Caf I or F1, 15.6 kDa) and the low calcium response antigen (LcrV or V, 37.2 kDa) have been identified as key components for vaccine design (116). F1 forms a capsule enabling host immune evasion, while V forms an injectisome pore to translocate virulence factors into host cells (116).

Although F1 is a good vaccine candidate, its tendency to aggregate negatively affects purification and vaccine efficacy, resulting in varied immune responses in humans (116). We have therefore constructed a mutant F1 antigen (F1mut) that cannot oligomerize and folds as a monomer (79). We have also fused the F1mut to the V antigen to produce a bivalent F1mut-V immunogen that is also a monomer in solution (79). The F1mut-V was then fused to the N terminus of Soc and arrayed on phage T4 nanoparticles by in vitro assembly. The T4-(F1mut-V-Soc) phage displaying about 663 F1mutV copies per capsid induced robust F1- and V-specific antibody responses in mice (79). Additionally, the T4-(F1mut-V-Soc) vaccine induced potent and balanced Th1 (cellular) and Th2 (humoral) immune responses, while the soluble F1mut-V with alum showed a strong bias toward Th2 responses and poor Th1 responses (79). Importantly, the T4-(F1mut-V-Soc) vaccine provided 100% protection to mice and Brown Norway rats (natural hosts for *Y. pestis*) against intranasal challenge with the highly lethal *Y. pestis* CO92 strain (79).

#### 4.3.3. The T4-plague prime-boost vaccine.

A single dose of the T4-plague prime-boost vaccine was tested using T4 heads packaged with the F1mut-V gene and displayed with Hoc-fused F1mut-V protein, enabling co-delivery in a single immunization (77). A single intramuscular immunization with these nanoparticles induced strong humoral and cellular responses in mice (77). Targeted F1mut-V DNA delivery into DCs resulted in the expression and presentation of the F1mut-V antigen for an extended period, leading to continuous stimulation of T cells (77).

In another study, the F1mut-V protein and AAV were co-displayed on the T4 head (80, 97). The ~25-nm AAV particle has the natural ability to enter mammalian cells and acts as an efficient driver for delivering the F1mut-V protein into cells (80). The T4-(F1mut-V-Soc)-AAV vaccine elicited higher levels of F1mut-V-specific antibody than T4-(F1mut-V-Soc) and provided complete protection against pneumonic plague in mice challenged with a high dose (295 LD_50_) of *Y. pestis* CO92 (80).

#### 4.3.4. The T4-plague-anthrax dual vaccine.

A dual vaccine against pneumonic plague and inhalation anthrax was developed by displaying the plague and anthrax antigens on the phage T4 capsid via Soc (22). Two proteins, F1mut-V-Soc (66 kDa) and Soc-PA (93 kDa), were displayed on the capsid in vitro with copy numbers of ~650 and 360, respectively (22). The resulting T4-plague-anthrax vaccine when administered either i.m. or i.n. induced robust F1-, V-, and PA-specific immune responses and generated balanced Th1- and Th2-based antibody responses, which are highly desirable for the clearance of pathogenic bacteria (22). This dual vaccine was highly effective in protecting animals against lethal challenges with both plague and anthrax agents in three different models: BALB/c mice, Brown Norway rats, and New Zealand White rabbits. Remarkably, the vaccinated animals were completely protected even when simultaneously challenged with anthrax lethal toxin and *Y. pestis* CO92 in the rat model or when exposed to inhalational anthrax via aerosolized Ames spores of *B. anthracis* in the rabbit model (22). These results suggest that the phage T4-plague-anthrax vaccine, particularly the nasal vaccine, has potential as a stockpiling candidate against bioterror attacks involving either one or both biothreat agents.

## 5. OTHER BACTERIOPHAGE VACCINE PLATFORMS

Many phage platforms have been widely studied for developing vaccines against infectious diseases and cancer due to their stability, biocompatibility, immunogenicity, low production costs, and flexible delivery methods (20, 45). During the pandemic, these were also adapted for designing COVID-19 vaccines (Table 2).

### 5.1. AP205 Phage

AP205, a single-stranded RNA phage targeting *Acinetobacter*, features a 27- to 30-nm icosahedral capsid enclosing a 4.3-kb genome. Its capsid, made of 180 coat protein units, has been used to display antigen epitopes fused to the N or C termini of the coat protein (117). The encapsulated RNA acts as a TLR-based adjuvant, enhancing both innate and adaptive immunity (118, 119). However, the AP205 nanoparticles alone show limited immunogenicity, necessitating an adjuvant such as Addavax or MF59 for stronger responses (118–121). The AP205-RBD vaccine, presenting about 72 copies of the 227-residue RBD per capsid, elicited protective immune responses in mice and rhesus macaques (118–120) and is currently in Phase II and III trials (NCT05077267; NCT05329220) to assess efficacy in humans.

### 5.2. λ Phage

The 60-nm-size icosahedral λ phage capsid is composed of 415 copies of the major capsid protein gpE and decorated with up to 420 copies of gpD protein. The gpD trimer stabilizes the capsid against the internal pressure of the 48.5-kb dsDNA genome (122). Capsids without gpD are unstable, making it an essential protein, but mosaic phages containing both wild-type gpD and gpD-antigen fusions are tolerated (123) and elicit effective immune responses in animal models (122). Davenport et al. (124) developed a λ-COVID vaccine by chemically attaching SARS-CoV-2 and MERS-CoV RBDs to gpD and displaying them on λ capsids. These capsids with 168 or 252 copies of RBDs induced strong nAbs and protected mice against SARS-CoV-2 and MERS-CoV challenges.

### 5.3. Qβ Phage

The 28-nm-size *E. coli* phage Qβ has a capsid with 180 A1 coat proteins and a 4.2-kb packaged RNA genome. It can display up to ~86 peptide epitopes fused to the coat protein or larger antigens chemically conjugated to capsid (125). Qβ VLPs have advanced to clinical trials for several infectious diseases, such as influenza, malaria, and HPV (45). To develop a COVID-19 vaccine, RBD was chemically conjugated to an engineered capsid up to ~20 copies per particle. This vaccine adjuvanted with monophosphoryl lipid A elicited RBD-specific antibodies with broad virus-neutralizing activity, robust cellular immunity including IFN-γ^+^ CD4^+^ and CD8^+^ T cell responses, and mucosal IgA responses (126, 127).

### 5.4. MS2 Phage

MS2, a RNA phage with a 26-nm capsid, can display peptides of up to 25 amino acids fused to its 180 copies of coat protein (128). For developing a COVID-19 vaccine, the capsid was decorated with ~18 SARS-CoV-2 spike trimers using the biotin-avidin system (129). A single dose of this Alhydrogel-adjuvanted vaccine produced nAbs and complete protection against SARS-CoV-2 in a hamster model.

### 5.5. Bxb1 Phage

The mycobacteriophage Bxb1 presents an icosahedral capsid with C-terminal extensions on its major capsid and tail proteins enabling peptide display (130). A 30-residue peptide of SARS-CoV-2 RBD fused to the C terminus of the capsid protein generated VLPs displaying ~400 copies of the peptide, but longer RBD sequences were not tolerated (131). These elicited weak and variable RBD-specific antibody responses, likely because the short RBD-30 segments deviated from the native conformation (131).

### 5.6. Filamentous Phages

Filamentous phages, ~900 nm long and 7 nm wide, contain an ~6.4-kb circular single-stranded DNA genome and are made of ~2,700 pVIII coat proteins and 5 copies each of four minor coat proteins, pIII and pVI at one end and pVII and pIX at the other end. Although all 5 proteins can display antigen peptides, pVIII is most commonly used due to its high copy number (132). The f88–4 filamentous phage was engineered for dual display of a SARS-CoV-2 spike epitope (10-residue, ~300 to 500 copies) and a lung-targeting peptide on pVIII and pIII (10-residue, 3 to 5 copies). This VLP vaccine elicited systemic antibody responses in mice when given intratracheally (133). However, filamentous phages are generally restricted to displaying short peptides on pVIII. Larger antigens, such as a 101-residue RBD P1 region on M13 phage, resulted in low copy numbers and weak immune responses (134).

## 6. CONCLUSIONS, LIMITATIONS, AND FUTURE PROSPECTS

The T4 phage is endowed with several desirable features that have been exploited in the past three decades to develop it as a protein-based, adjuvant-free vaccine design platform (20, 23). Among the key features, from the pandemic standpoint, are its remarkable stability that likely preserves the conformational integrity of antigens displayed, its ability to stimulate robust systemic and mucosal responses, and its amenability for needle-free nasal delivery. Its large-capacity capsid with a symmetrical bio-lattice enables high-density display of antigens, from short peptides to large proteins and oligomeric complexes, in a plug-and-play, multivalent display format. Its apparent affinity to nasal mucosa through the naturally evolved Hoc allows persistence in the respiratory tract triggering sIgA and T_RM_ responses that can confer sterilizing immunity against respiratory pathogens (21, 81).

**Figure 5:**
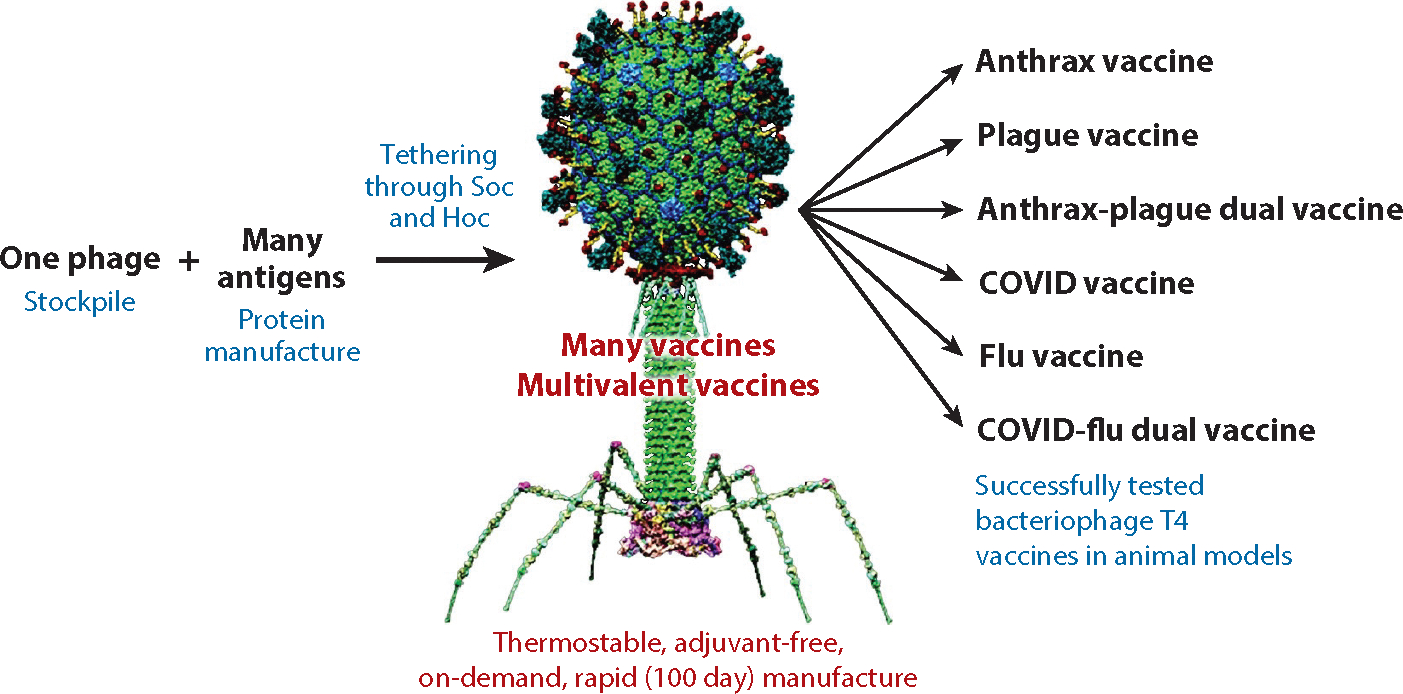
Bacteriophage T4 as a pandemic mucosal vaccine design platform for rapid production of monovalent and multivalent vaccines. Utilizing stockpiled T4 phage and tethering with any antigen(s) of interest, thermostable vaccines can be rapidly produced and distributed across the globe in the event of an epidemic or pandemic.

While other intranasal vaccine platforms are emerging, most employ live or live-attenuated eukaryotic viruses such as the adenoviruses (100, 104), influenza viruses (135), vesicular stomatitis virus (136), Newcastle disease virus (137), and so on. However, preexisting immunity from natural infections or widespread vaccinations such as Ad-COVID-19 might limit their effectiveness (11, 138). The noninfectious T4 vaccine platform provides an excellent alternative, and it can be inexpensively and rapidly manufactured and distributed without a cold-chain requirement (21, 23), even in remote areas of the world. It is thus possible to establish a 100-day, protein-based, T4 mucosal vaccine production platform in the event of a pandemic, which can complement the mRNA and DNA (adenoviral) vaccine platforms (Figure 5). The phage scaffold can be stockpiled, and any pandemic antigen(s) can be rapidly produced in bacterial, insect cell, or mammalian cell expression systems and loaded on the capsid by simple mixing of the two to create a variety of phage nanoparticle vaccine(s).

Despite these useful features, T4 (and phages in general) remains an early-stage platform. Transitioning this platform from preclinical to clinical stages requires substantial additional funding and resources. First, streamlined GMP processes for large-scale phage production, purification, and quality control including low and consistent endotoxin levels need to be established. Progress is being made in this regard through the production of GMP-quality phages for antibacterial phage therapy applications (139, 140). Second, extensive testing in animal and nonhuman primate models is still needed to fully evaluate safety, biodistribution, and pharmacokinetics for vaccine and clinical applications (141). Third and perhaps most importantly, the government and nonprofit organizations and biotechnology industry ought to realize that it is in strategic public interest to invest now in new and potent vaccine platforms to effectively mitigate complex pandemics in the future. T4 and other phages have been well demonstrated as a robust vaccine platform against diverse pathogens.

In the current environment, however, funding and attention are largely directed to further development and expansion of the well-established mRNA and adenoviral platforms rather than investing in new and complementary next-generation platforms. While this might reap dividends in the short run, it might not be so in the long run, given the unpredictability of future pandemics and the potential new challenges they might pose. Of particular importance are the platforms that offer thermostability, rapid and inexpensive manufacture, and needle-free administration. Such a strategic approach is vital not only for robust pandemic preparedness but also for ensuring equitable vaccine access to low-income communities worldwide, who continue to face challenges in obtaining the COVID-19 vaccines (142, 143).

## Figures and Tables

**Table 1:** Summary of key features and findings for various vaccine candidates engineered using the T4 bacteriophage platform

Vaccine (route)	Antigens	Capsid protein used	Immune responses			Animal mod el (s) tested	Protection efficacy
			Humoral	Cellular	Mucosal		
T4-COVID (i.m. and i.n.)	Spike trimer; Ee epitope; NP	Hoc, Soc	High IgG and IgA titers; balanced IgG1 and IgG2a; neutralizing antibodies against MA10, WA-1, Beta, Delta, Omicron	Increased IFN-γ^+^ CD4^+^/CD8^+^ T cells; increased Th1 (IFN-γ, IL-2, TNF-α),Th2 (IL-4), Thl 7 (IL-17A) cytokines	Potent IgG and slgA in BALF (i.n.)	Mouse, rabbit	Complete protection against MA10, WA-1, Delta SARS-CoV-2, and VOC strains
T4-Flu (i.m. and i.n.)	3M2e (human, swine, and avian); HA stem domain	Soc	High M2 e-specific IgG titers; balanced IgGl/IgG2a	Increased M2e-specific IFN-γ^+^ and IL-4^+^ splenic T cells; M2e-specific lung T_EM_ and T_RM_ cells (i.n.)	M2 e-specific IgG and slgA in BALF (i.n.)	Mouse	Complete protection against H1N1 and H3N2 influenza strains (i.n.)
T4-Anthrax (i.m.)	PA; LF; EF; toxin complexes	Hoc, Soc	High IgG and lethal toxin-neutralizing antibody titers; balanced IgG1/IgG2a	Not tested	Not tested	Mouse, rabbit, macaque	Complete protection against inhalational anthrax spore challenge
T4-Plague (i.m.)	Flmut-V (protein and/or DNA)	Soc	Strong anti-F1 and anti-V IgG titers; balanced IgG1/IgG2a	Increased IFN-γ^+^ splenic T cells	Not tested	Mouse, rat, rabbit	Complete protection against intranasal *Y. pestis* CO92 challenge
T4-Plague-Anthrax (i.m.)	Flmut-V and PA	Soc	Potent F1-, V-, PA-specific IgG; balanced IgG1/IgG2a	Not tested	Not tested	Mouse, rat, rabbit	Complete survival against simultaneous *Y. pestis* and lethal toxin challenges

The vaccines elicited potent and broad systemic and mucosal immune responses and provided complete protection against the target pathogens.

Abbreviations: BALF, bronchoalveolar lavage fluid; Ee, external domain of envelope protein; EF, edema factor; HA, haemagglutinin; Hoc, highly antigenic outer capsid protein; i.m., intramuscular; i.n., intranasal; LF, lethal factor; M2e, matrix protein 2; NP, nucleocapsid protein; PA, protective antigen; sIgA, secretory IgA; Soc, small outer capsid protein; T_EM_, effector memory T cell; T_RM_, tissue-resident memory T cell; VOC, variant of concern.

**Table 2:** Comparison of various phage platforms for COVID-19 vaccine development

	T4	AP205	*λ*	Qβ	MS2	Bxb1	f88-4
Capsid size, nm	120 × 86	27–30	60	28	26	50	~900 ×−7
Genome	~171-kbp dsDNA	~4.3-kb ssRNA	48.5-kbp dsDNA	~4.2-kb ssRNA	~3.6-kb ssRNA	50-kbp dsDNA	~6.4-kb ssDNA
Phage protein for display (eopies/capsid)	Hoc (155); Soc (870)	CP (180)	gpD (405–420)	A1 (3–86)	CP (180)	CP (415)	pVIII (2,700); pill(5)
Preferred molecule for in vivo display (maximum copies)	Full-length protein; peptide (1,025)	Up to 55 aa peptide (180)	Peptide (420)	Peptide (86)	Up to 2 5 aa peptide (180)	Up to 30 aa peptide (415)	Peptide (2,700)
COVID-19 antigen display method	Fusion to Hoc, Soc, and IP3-CTS; S pyCatcher-S pyTag	Fusion to CP via S pyCatcher- SpyTag	Chemical cross-link to gpD	Chemical conjugate	Avidin-biotin linkage	Fusion to CP	Fusion to pVIII
Copy number COVID-19 antigen displayed	~100 copies spike trimer; ~150 copies Ee epitope; ~100–150 copies NP	~72 copies RBD	168–252 copies RBD	~20 copies RBD	~18 copies spike trimer	~400 copies RBD peptide (30 aa)	~300–500 copies spike epitope (10 aa)
COVID-19 animal model/human trial	Mice; rabbit	Mice; NHPs; human Phase I	Mice	Mice	Hamster	Mice	Mice
Adjuvant and administration routes	No adjuvant, in PBS; i.m. or i.n.	AddaVax or MF59; i.m.	No adjuvant; i.m.	MPLA; s.c.	AH; s.c.	No adjuvant; i.p.	No adjuvant; i.t.
Humoral response	IgG and IgA; balanced IgG1 and IgG2a; nAb	IgG; IgG2a/c biased; nAb	IgG; nAb	IgG	IgG; nAb	Weak and variable IgG	IgG
Cellular response	IFN-γ^+^ CD4^+^/CD8^+^ T cells; balanced Th1/Th2/Th17 responses	IFN-γ^+^ CD4^+^ T cells	ND	IFN-γ^+^ CD4^+^/CD8^+^ T cells	ND	ND	ND
Mucosal response	IgA, IgG, and nAb in BALF	ND	ND	IgA and IgG in BALF	ND	ND	ND
Protection	Complete; no virus detected in lungs	Partial protection in NHPs	Complete; no virus detected in lungs	ND	Complete; no virus detected in lungs	ND	ND

Different phage platforms that have been engineered to display SARS-CoV-2 antigens and tested for immune responses and protection against COVID-19 were compared.

Abbreviations: aa, amino acid residue; AH, Alhydrogel; BALF, bronchoalveolar lavage fluid; CP, capsid protein; CTS, capsid targeting sequence; ds, double-stranded; Ee, external domain of envelope protein; i.m., intramuscular; i.n., intranasal; i.p., intraperitoneal; i.t., intratracheal; MPLA, monophosphoryl lipid A; nAb, neutralizing antibody; ND, not determined; NHP, nonhuman primate; NP, nucleocapsid protein; PBS, phosphate-buffered saline; RBD, receptor binding domain; s.c., subcutaneous; Soc, small outer capsid protein; ss, single-stranded.
